# Changes and Exchanges

**Published:** 1855-07

**Authors:** 


*Changes and Exchanges.*—Dr. Horace Green resigns the chair of Practice of Medicine in the New York Medical College, and Dr. H. G. Coxe succeeds him.

Dr. Brown Sequard has very suddenly resigned the chair of Physiology and Pathology in the Virginia Medical College. Dr. Joynes assumes the vacant place. Dr. Peticolas, of Richmond, takes the chair of Anatomy, vacated by the loss of Prof. Carter Johnson in the Arctic.

Dr. Henry H. Smith succeeds Prof. Gibson in the chair of Surgery, at University of Pennsylvania. The vote in the board of trustees stood 11 for Dr. Smith, 9 for Dr. Brainard, of Chicago, and 1 blank. It is understood that Dr. Brainard was the candidate of the faculty.

Among our exchanges we have to note the accession of Dr. C. D. Griswold to the American Medical Gazette, in association with Dr. Reese; the abandonment of the project of a medical quarterly at New Orleans, to be edited by Dr. Bennett Dowler; and the appearance of a new weekly journal —the Medical Counsellor—at Columbus, Ohio, edited by R. Hill, M. D.

The New Orleans Medical News and Hospital Gazette, is the title of a new journal in New Orleans, edited with much spirit, tact, and judgment, by Drs. Choppin, Beard, and Vance.

Finally, Dr. Richard Gundry becomes the assistant editor of the Ohio Medical and Surgical Journal. Dr. Gundry is a gentleman of very high professional attainment, an easy, graceful writer, and cannot fail to make a good editor.

